# New Bills of Mortality Tables for London

**Published:** 1842-04

**Authors:** 


### PART FOURTH.

### Medical Intelligence.

### TABLE OF MORTALITY FOR LONDON,

For the Fourth Quarter of 1841: Showing the number of Deaths from all causes registered in 14 weeks, from Sept. 25, 1841, to Jan. 1, 1842; also for the whole year 1841.

| Causes of Death. | Total Deaths. During the Quarter. | Total Deaths. During the Year. |
|---|---|---|
| **Class I.** | | |
| Smallpox .. | 68 | 1053 |
| Measles .... | 408 | 973 |
| Scarlatina .. | 180 | 663 |
| Hooping-cough | 634 | 2 278 |
| Croup ...... | 126 | 391 |
| Thrush .... | 61 | 260 |
| Diarrhœa .... | 110 | 465 |
| Dysentery .. | 20 | 78 |
| Cholera .... | 3 | 28 |
| Influenza .... | 13 | 220 |
| Ague........ | 3 | 15 |
| Remit. Fever . | 1 | 16 |
| Typhus .... | 288 | 1151 |
| Erysipelas .. | 64 | 251 |
| Syphilis .... | 7 | 29 |
| Hydrophobia . | 2 | 3 |
| Total .... | 1988 | 7874 |
| **Class II.** | | |
| Cephalitis.... | 121 | 615 |
| Hydrocephalus | 374 | 1739 |
| Apoplexy .... | 231 | 866 |
| Paralysis .... | 207 | 751 |
| Convulsions .. | 572 | 2778 |
| Tetanus .... | 3 | 20 |
| Chorea ...... | 1 | 6 |
| Epilepsy .... | 41 | 181 |
| Insanity .... | 11 | 43 |
| Delir. tremens | 27 | 83 |
| Disease ...... | 101 | 478 |
| Total .... | 1689 | 7560 |
| **Class III.** | | |
| Laryngitis .. | 6 | 27 |
| Quinsy ...... | 20 | 71 |
| Bronchitis .. | 135 | 665 |
| Pleurisy .... | 29 | 93 |
| Pneumonia .. | 931 | 3668 |
| Hydrothorax . | 45 | 208 |
| Asthma .... | 291 | 1351 |
| Consumption . | 1672 | 7326 |
| Disease ...... | 175 | 768 |
| Total .... | 3304 | 14177 |

| Causes of Death. | Total Deaths. During the Quarter. | Total Deaths. During the Year. |
|---|---|---|
| **Class IV.** | | |
| Pericarditis .. | 3 | 30 |
| Aneurism .... | 6 | 36 |
| Disease .... | 237 | 927 |
| Total ...... | 246 | 993 |
| **Class V.** | | |
| Teething .... | 227 | 913 |
| Gastritis enter. | 215 | 957 |
| Peritonitis .. | 13 | 59 |
| Tabes mesen. | 73 | 261 |
| Worms ...... | 5 | 23 |
| Ascites ...... | 13 | 31 |
| Ulceration .. | 20 | 74 |
| Hernia ...... | 25 | 103 |
| Colic or ileus . | 33 | 130 |
| Intussusceptio. | 3 | 14 |
| Stricture .... | 4 | 26 |
| Hæmatemesis | .. | 11 |
| Dis. of stomach | 26 | 179 |
| Dis. of pancreas | 1 | 1 |
| Hepatitis .... | 6 | 59 |
| Jaundice .... | 34 | 111 |
| Dis. of liver .. | 110 | 436 |
| Dis. of spleen . | .. | 2 |
| Total .... | 808 | 3390 |
| **Class VI.** | | |
| Nephritis .... | 8 | 26 |
| Ischuria .... | 1 | 7 |
| Diabetes .... | 2 | 18 |
| Cystitis...... | 5 | 12 |
| Stone ...... | 3 | 17 |
| Stricture .... | 4 | 15 |
| Disease...... | 29 | 139 |
| Total .... | 52 | 234 |
| **Class VII.** | | |
| Childbirth .. | 88 | 345 |
| Paramenia .. | 3 | 10 |
| Ovarian dropsy | 1 | 18 |
| Disease .... | 20 | 137 |
| Total...... | 112 | 510 |

| Causes of Death. | Total Deaths. During the Quarter. | Total Deaths. During the Year. |
|---|---|---|
| **Class VIII.** | | |
| Arthritis .... | .. | 2 |
| Rheumatism . | 28 | 120 |
| Disease .... | 28 | 129 |
| Total...... | 56 | 251 |
| **Class IX.** | | |
| Carbuncle.... | .. | 1 |
| Phlegmon .. | 3 | 5 |
| Ulcer ...... | 10 | 24 |
| Fistula .... | 3 | 13 |
| Disease .... | 1 | 12 |
| Total...... | 17 | 55 |
| **Class X.** | | |
| Inflammation | 65 | 238 |
| Hemorrhage.. | 28 | 165 |
| Dropsy .... | 450 | 1720 |
| Abscess .... | 44 | 169 |
| Mortification | 54 | 241 |
| Purpura .... | 2 | 12 |
| Scrofula .... | 22 | 105 |
| Carcinoma .. | 93 | 373 |
| Tumour .. | 31 | 100 |
| Gout ...... | 12 | 61 |
| Atrophy .... | 78 | 363 |
| Debility .... | 290 | 1114 |
| Malformations | 5 | 36 |
| Sudden deaths | 191 | 759 |
| Total...... | 1365 | 5456 |
| **Class XI.** | | |
| Old age .... | 778 | 3373 |
| **Class XII.** | | |
| Intemperance | 3 | 30 |
| Privation .. | 9 | 36 |
| Violent deaths | 284 | 1148 |
| Total .... | 296 | 1214 |
| **Class XIII.** | | |
| Not specified | 50 | 197 |

| | For the Quarter. | | For the Year. |
|---|---|---|---|
| Total Deaths from all causes (Males) .... | 5455 | .... | 22,995 |
| „ „ (Females) .. | 5306 | .... | 22,288 |

### TEMPERATURE DURING THE QUARTER AND YEAR.

| | For the Quarter. | | For the Year. |
|---|---|---|---|
| Highest ...................... | 63 7 | .... | 87·0 |
| Lowest ...................... | 28·2 | .... | 14·9 |
| Daily mean ................. | 46·4 | .... | 51·7 |

